# The characteristics of patients requiring readmission to an Australian forensic psychiatric intensive care unit

**DOI:** 10.1192/j.eurpsy.2021.1009

**Published:** 2021-08-13

**Authors:** T. Jones, E. Harris, M. Roberts, D. Mawren, S. Lee

**Affiliations:** 1 Psychiatry, Victorian Institute of Forensic Mental Health, Melbourne, Australia; 2 Centre For Forensic Behavioural Science, Swinburne University of Technology, Melbourne, Australia

**Keywords:** Forensic, psychiatry, PICU

## Abstract

**Introduction:**

Psychiatric intensive care units (or PICU’s) emerged to manage high acuity patients outside the justice system. Studies have sought to better understand characteristics of those admitted to forensic or civilian PICU’s. Few, in contrast, have explored the frequency and contributors to readmission. The following study was conducted on Apsley unit, a Forensic PICU based in Melbourne, Australia, and seeks to understand the differences which would allow early identification of patients likely to require readmission and the provision of targeted interventions.

**Objectives:**

Examine rates of and contributors to forensic PICU readmission over a 6-month period.

**Methods:**

A retrospective audit was conducted to collect clinical, problem behaviour (and strategies to manage), forensic history and demographic information for consecutively admitted patients to an 8-bed forensic PICU between March-September 2019.

**Results:**

Data analysis is ongoing. Interim analysis found that 96 patients were admitted during the 6-month study period: 74 (77.1%) had a single admission; 22 (22.9%) required readmission. Almost all were admitted from prison (96.9%), most had a psychosis diagnosis (80.2%) and substance abuse history (96.9%), and many had a personality disorder (24.0%) and history of adolescent antisocial behaviour (46.5%). Patients requiring readmission were significantly more likely to have been previously under compulsory mental health treatment (95.5% vs 75.3%, p=.039) and have a Positive Behaviour Support Plan developed during admission (85.7% vs 54.8%, p=.010).

**Conclusions:**

Interim analysis highlighted the multicomplexity for forensic PICU patients alongside the occurrence of problem behaviour during admission and history of compulsory treatment as indicators of increased risk for re-admission.

